# High-Frequency Miniprobe Endoscopic Ultrasonography Across the Gastrointestinal Tract

**DOI:** 10.3390/diagnostics16091316

**Published:** 2026-04-28

**Authors:** Francesco Bombaci, Angelo Bruni, Margherita Pavanato, Giuseppe Dell’Anna, Francesco Vito Mandarino, Giulio Calabrese, Andrea Lisotti, Pietro Fusaroli, Leonardo Henry Eusebi, Giovanni Barbara, Paolo Cecinato

**Affiliations:** 1 Department of Medical and Surgical Sciences, University of Bologna, 40138 Bologna, Italy; francesco.bombaci@studio.unibo.it (F.B.); margherita.pavanato@studio.unibo.it (M.P.); pietro.fusaroli@unibo.it (P.F.); giovanni.barbara@unibo.it (G.B.); 2 Gastroenterology Unit, IRCCS Azienda Ospedaliero-Universitaria di Bologna, 40138 Bologna, Italy; 3 Hôpital Prive Armand Brillard, CEI Centre Endoscopie Interventionnelle, 94130 Paris, France; 4 Gastroenterology and Gastrointestinal Endoscopy Unit, IRCCS San Raffaele Hospital, 20132 Milan, Italy; dellanna.giuseppe@hsr.it (G.D.); mandarino.francesco@hsr.it (F.V.M.); 5 Gastroenterology and Gastrointestinal Endoscopy Division, IRCCS Policlinico San Donato, 20097 San Donato Milanese, Italy; 6 Gastroenterology Unit, San Pio Hospital, 82100 Benevento, Italy; 7 Department of Clinical Medicine and Surgery, University of Naples Federico II, 80131 Naples, Italy; 8 Gastroenterology Unit, Hospital of Imola, University of Bologna, 40026 Imola, Italy; lisotti.andrea@gmail.com

**Keywords:** miniprobe endoscopic ultrasonography, high-frequency ultrasound catheter probes, intraductal ultrasonography, endoscopy ultrasonography

## Abstract

Miniprobe endoscopic ultrasonography (mEUS) combines high-resolution imaging of the gastrointestinal (GI) wall and bile ducts with ease of applicability during routine endoscopy. This narrative review aims to provide an overview of known and emerging fields of application for mEUS in gastrointestinal endoscopy. After its initial development in pancreatobiliary scenarios in the early 1990s, mEUS has been recently reconsidered a third-space endoscopic technique that is progressively developing and spreading for the treatment of early gastrointestinal neoplastic lesions. The high spatial resolution of mEUS provides an accurate assessment of the degree of submucosal invasion in early esophageal, gastric, and colorectal neoplasia, while the small caliber of catheters allows for mEUS employment in settings where standard echoendoscopes are impractical (e.g., severe stenoses or proximal colonic lesions). Beyond cancer staging, mEUS offers point-of-care characterization of subepithelial lesions by defining the layer of origin and echo-pattern, eventually defining endoscopic resectability, but definitive diagnosis remains histological. In pancreatobiliary diseases, miniprobe intraductal ultrasonography (IDUS) shows its strongest application for indeterminate biliary strictures when endoscopic retrograde cholangiopancreatography (ERCP)-based sampling strategies and brushing cytology show inconclusive diagnoses, and in choledocholithiasis, particularly for the detection of small stones/sludge and confirmation of duct clearance. IDUS is also valuable for the staging of ampullary tumors, for longitudinal extension mapping in hilar cholangiocarcinoma and for selected portal biliopathy scenarios. Overall, mEUS and IDUS are high-resolution adjuncts that can meaningfully refine local decision-making in the treatment of superficial epithelial/subepithelial tumors or lesions involving the bile ducts. Limitations include shallow penetration, lack of tissue acquisition capability, a relative increase in post-ERCP pancreatitis risk for intraductal use, and substantial cost with limited availability in lower-volume centers.

## 1. Introduction

Miniprobe endoscopic ultrasonography (mEUS) refers to the use of high-frequency catheter probes that provide high-resolution imaging of the gastrointestinal (GI) wall. When employed for the study of biliary ducts and perivisceral structures, it is also known as “intraductal ultrasonography” (IDUS). Miniprobes were developed in the early 1990s, when technological advances resulted in a significant reduction in the size of ultrasound transducers, in order to overcome some limitations of standard EUS [1].

**Figure 1:**
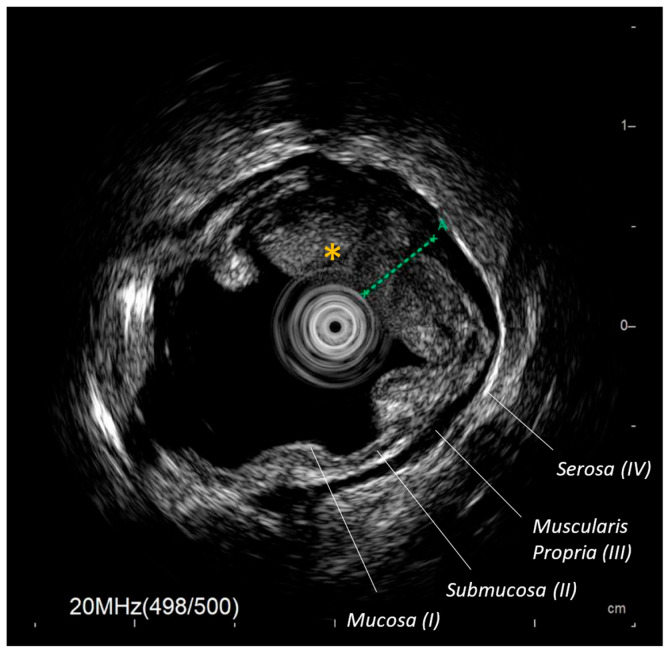
Example of a pre-operative mEUS image of an early colorectal cancer (*) of the right colon, obtained with a 20 MHz miniprobe (iMP-8903, InnerMed). The image shows tumor involvement of the mucosa and deep submucosa (dashed line), with initial invasion of the muscularis propria (layer III, hypoechoic). The patient underwent right hemicolectomy, and final histology confirmed locally advanced disease (pT4N1).

Typically 2–3 mm in diameter, miniprobes are introduced through the working channel of standard endoscopes and can traverse severe strictures, where standard echoendoscopes are limited because of larger diameter (12–13 mm) [2]. The longest mEUS model reaches 2700 mm in length, making it suitable also for enteroscopes [3]. The mEUS scanning method may be mechanical or electronic. Mechanical miniprobes have a rotating ultrasound transducer housed within a plastic sheath at the tip of the probe, while electronic miniprobes have a ring at the tip of the probe with 64 transducer elements that produce a 360° radial scan with images comparable to those from a standard radial EUS [4]. Some newer systems even offer dual-plane (forward viewing), miniature linear scanning and optional 3D reconstruction, though radial scanning is the most used [5]. Acoustic coupling with the area of interest may be hindered by artifacts (e.g., air bubbles, stool), but they can be limited by filling the organ lumen with water or by using a balloon sheath on the probe tip [6,7]. Currently, mEUS models available on the market operate at frequencies ranging from 7.5 MHz to 30 MHz. Higher-frequency probes (e.g., 20–30 MHz) provide excellent spatial resolution (0.07–0.18 mm), which makes them particularly suitable for the assessment of superficial lesions of the GI wall’s layers (mucosa, submucosa, muscularis propria, serosa) or for the intraductal evaluation of mural thickening and subtle intraluminal lesions of biliary ducts (Figure 1). On the other hand, they have a limited penetration depth of 1.5–2.0 cm, whereas lower frequencies (12 MHz or below) can penetrate up to 3 cm with slightly lower resolution [8]. Furthermore, mEUS does not have a Doppler function, but intravenous injection of a contrast agent may enhance vascularization [9].

Table 1 summarizes the technical features of most miniprobe models currently available on the market.

Unlike standard echoendoscopes, miniprobes have limited durability (60–100 uses). Moreover, mEUS is a purely diagnostic imaging tools, as these catheter probes are not built with a working channel for biopsy or needle intervention [5]. On the other hand, mEUS has been recently employed to guide interventional procedures in the bile ducts [10,11], and its “on-demand” approach, immediately upon detecting a lesion during routine endoscopy, eliminates the need for a separate standard EUS exam [11].

Early clinical experience suggested that adding mEUS during conventional endoscopy provided relevant supplementary information in approximately 70% of examinations and influenced therapeutic decisions in over half of patients in some series [5,12]. Moreover, mEUS has a safety profile similar to conventional endoscopy, with the exception of a moderate increase in the risk of post-procedural pancreatitis for intraductal biliary applications [13]. A brief comparison between mEUS/IDUS and standard EUS/cross-sectional methods is presented in Table 2.

Despite these advantages, the clinical role of mEUS remains heterogeneous and strongly indication-dependent; its main limitations include shallow penetration depth, lack of tissue acquisition capability, and limited performance for comprehensive nodal or extramural assessment. Contemporary reviews emphasize its role as a rapid “point-of-care” adjunct—particularly for early carcinomas in selected scenarios, gastrointestinal subepithelial lesions (SELs), and conditions where standard EUS is impractical—rather than a replacement for standard EUS, particularly when comprehensive nodal staging or tissue acquisition is required [8]. Currently, major Western guidelines do not issue specific recommendations or formal indications for mEUS [14,15,16,17,18]. However, ongoing technical probe innovation (e.g., photoacoustic endoscopic probes integrating optical excitation with ultrasound detection) may further expand the diagnostic scope of mEUS endoluminal imaging in the future [19].

The aim of this review is to summarize the technical principles, validated applications, diagnostic performance and limitations of mEUS across major GI indications, with a practical focus on comparison with standard EUS and cross-sectional imaging in everyday decision-making.

## 2. Materials and Methods

This article was conceived as a narrative review informed by a structured literature search. To improve methodological transparency, the search framework, eligibility criteria, and study selection process are summarized in Table 3. Given the broad clinical scope of the review and the heterogeneity of indications, study designs, ultrasound platforms, comparator standards, and reported endpoints, the available evidence was synthesized qualitatively and organized by anatomical district and clinical scenario, including oncologic staging, luminal strictures, subepithelial lesions, and selected benign applications. Where available, data extraction focused on study design, population size, probe frequency and technical platform, feasibility, diagnostic performance parameters, comparisons with standard EUS or cross-sectional imaging, and adverse events. No quantitative meta-analysis or formal risk-of-bias assessment was performed because of the narrative design of the study and the marked heterogeneity of included studies. Accordingly, readers should interpret the findings cautiously, as a clinically oriented synthesis of the available literature, contextualized against major society guidelines.

## 3. Clinical Applications of mEUS Across Esophageal, Gastric and Colorectal Disorders

### 3.1. Esophageal Cancer and Barrett’s Esophagus-Associated Lesions

**Figure 2:**
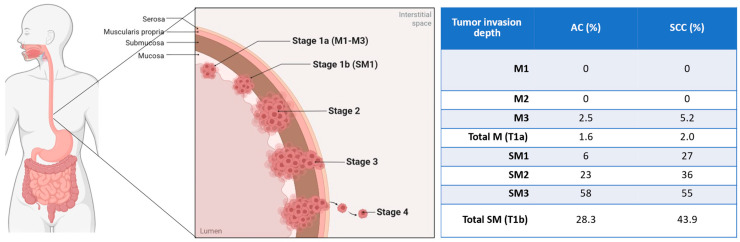
Risk of lymph node metastasis for early esophageal cancer according to histology and tumor invasion depth. SCC—squamous cell carcinoma; AC—adenocarcinoma.

EUS can be employed for the staging of esophageal cancer without distant organ metastases, as it helps determine the tumor (T) category and locoregional lymph node (N) involvement [20]. Previous studies have shown that EUS staging significantly impacts the multidisciplinary management strategy of patients with esophageal cancer, supporting a more appropriate use of preoperative neoadjuvant therapy [21] and refining the selection of high-risk esophagectomy towards non-surgical palliation [22]. For early esophageal cancer, the European Society of Gastrointestinal Endoscopy (ESGE) suggests performing EUS when suspicious endoscopic features for deep submucosal invasion are present [17]. However, standard EUS has known limitations in reliably staging very early (T1) esophageal tumors [23,24,25], where precise discrimination between mucosal (M) and submucosal (SM) invasion is required to select the type of endoscopic treatment or surgical therapy [17]. This distinction is clinically relevant because lymph node metastasis rates are markedly depth-dependent, with a higher risk for squamous cell carcinoma than for adenocarcinoma [26,27] (Figure 2).

In this setting, mEUS allows high-resolution assessment of the esophageal wall, thereby contributing to the evaluation of invasion depth in early esophageal cancer. Initial evidence on mEUS showed an overall accuracy of 87% for T-staging, particularly for distinguishing M1-M2 from M3 invasion depth (93% accuracy) [28]. This is relevant because M3 disease for esophageal squamous cell carcinoma represents the transition between very low-risk intramucosal disease and a lesion with a clinically meaningful risk of lymph node metastasis, where additional staging or treatment is generally recommended [17]. Between the late 1990s and early 2000s, several groups investigated the diagnostic performance of mEUS for esophageal cancer staging, with heterogeneous results but a consistent pattern: stronger T-staging than N-staging. In the early surgical series of Hünerbein et al. [29], mEUS achieved high accuracy for determining tumor invasion depth (90%), whereas nodal assessment was suboptimal (78%) and constrained by limited ultrasound penetration depth (<3 cm) in bulky or locally advanced tumors. More modest results emerged from the study of Chemaly et al. [30], who employed mEUS for preoperative staging of esophageal cancers before endoscopic mucosal resection (EMR) or surgery, with an overall accuracy of 74% (61.9% sensitivity and 76.5% specificity) for detecting tumor submucosal invasion. Interestingly, the accuracy was not influenced by the histological subtype (squamous cell carcinoma or Barrett-related adenocarcinoma) [30]. On the other hand, in a more recent multicenter study including a larger cohort of patients, mEUS proved to have a higher specificity in differentiating locally advanced from early esophageal cancer (97% for T1, 84% for T2, 82% for T3–T4 disease) [31]. Consequently, 78% of the patients would have been assigned to the adequate therapeutic regimen [31]. However, for both studies, accuracy was lower near the esophagogastric junction and the rate of mis-staging played a significant role [30,31].

Direct comparisons between mEUS and standard EUS further clarified this point, but heterogeneity was still present among results. According to the retrospective studies of Hasegawa et al. [32] and Hünerbein et al. [12], mEUS was generally more accurate (87–92%) for T-staging rather than standard EUS (76–81%) and was clearly superior for the detection of T1 tumors (81% vs. 56%) [12], whereas N-staging remained modest and variable for both approaches (mEUS sensitivity 25%, specificity 80%; standard EUS sensitivity 50%, specificity 80%) [32]. By contrast, in the comparative study of Nesje et al. [33], mEUS and standard EUS showed similar accuracy (87%) for T-staging, but mEUS could not reliably discriminate T3 from T4 due to limited ultrasound penetration. Again, N-staging favored standard EUS (78% vs. 61% accuracy), especially when the tumor was not stenotic [33]. However, despite the lower performance for N-staging, mEUS can still provide a high negative predictive value for excluding locoregional lymph node metastasis (84%) [34]. Finally, mEUS has been compared with standard EUS for Barrett’s-related esophageal adenocarcinoma, showing only moderate accuracy for both approaches (69% vs. 76%, respectively) [35]. Actually, EUS-based T-staging rarely changes the therapeutic strategy because, for visible lesions, endoscopic resection provides the most accurate T-staging and allows assessment of prognostic factors not captured by EUS (e.g., lymphovascular invasion) [36]. As a consequence, current ESGE guidelines on Barrett-related neoplasia advise complete staging with EUS to detect locoregional lymph node metastasis only after endoscopic resection revealing T1b disease [37].

Overall, the evidence supporting mEUS for locoregional staging of early esophageal cancer remains limited across studies and is largely derived from small, single-center cohorts with variable inclusion criteria. Moreover, even if mEUS showed discrete accuracy for discriminating between M- and SM-invasion, endoscopic resection remains the preferred approach for definitive T-staging and for the assessment of non-EUS-determinable high-risk features, which ultimately drive the need for additional therapy. Accordingly, mEUS should be considered an on-demand tool in selected cases with macroscopic suspicion for deep submucosal invasion [38].

A summary of the current evidence is reported in Table 4.

Since miniprobes can traverse tight strictures, mEUS has been used to assess locoregional staging in near-obstructing esophageal cancer [39]. Before the introduction of mEUS, the only way to assess stenotic esophageal cancer with standard EUS was to previously dilate the stricture in order to allow the passage of a standard echoendoscope, but this procedure carries a risk of perforation of up to 24% [40,41]. In a comparative study between mEUS and standard EUS, Mennigen et al. [39] showed that mEUS has an acceptable accuracy for T-staging (73%) and N-staging (74%) of esophageal stenotic tumors that preclude the passage of a standard EUS probe. Therefore, mEUS can avoid performing previous dilatation in this group of patients, with similar diagnostic performance to standard EUS for non-stenotic esophageal tumors within the same cohort [39]. Furthermore, mEUS can provide more precise data on tumor longitudinal extension and invasion depth, which is prognostically meaningful, particularly for advanced esophageal squamous cell carcinoma requiring chemotherapy. In fact, a tumor length of <6 cm has been shown to predict better survival and a higher rate of downstaging after initial chemoradiotherapy. Moreover, when a tumor is re-staged post-chemoradiotherapy, a wall thickness of < 8 mm has been suggested as having the best operating characteristics for predicting 1-year survival [42]. Prognostic stratification further improves when mEUS response criteria are combined with CT assessment of metastatic progression, underscoring its complementary role with cross-sectional imaging for systemic disease evaluation [42].

### 3.2. Benign Esophageal Strictures and Other Benign Esophageal Diseases

Benign esophageal strictures are usually secondary to peptic disease, endoscopic treatment/surgery, caustics, radiotherapy, drugs, or eosinophilic esophagitis, and the mainstay of therapy for benign esophageal stenotic lesions is endoscopic dilation [43,44]. Previous studies suggested that in patients with benign esophageal strictures, EUS can provide detailed information about the esophageal wall, including the wall thickness and the extent of fibrosis [45]. These characteristics are particularly relevant because they influence stricture severity and the response to endoscopic dilation [46]. However, incomplete EUS examinations of the stricture were a limitation of these studies, as the esophageal strictures included were not negotiable with standard echoendoscopes; thus, only the proximal end of the narrowing could be evaluated, with potential understaging [45]. In this setting, a retrospective study showed that mEUS was be able to traverse tight benign esophageal strictures and provide a complete characterization of the esophageal wall thickness and the depth of fibrotic involvement across wall layers. Even though only 24 patients were included in the study, it confirmed that these characteristics possess particular prognostic relevance as the depth of fibrotic involvement, particularly of muscularis propria, predicted a higher dilatation burden and symptomatic recurrence [47]. On the other hand, when the histology of the stricture is still to be determined, a thicker esophageal wall and the loss of wall stratification have been more commonly associated with malignancy [48]. In the pediatric setting, miniprobes can also traverse congenital esophageal stenosis and achieve high diagnostic concordance with histopathology/intraoperative findings, thereby influencing the selection between dilation and surgery [49].

The application of EUS in esophageal motility diseases has not been well established, and current guidelines actually do not suggest its use in this field [50,51,52]. However, previous studies revealed a possible role for EUS in excluding pseudoachalasia caused by malignancy or as a useful adjunct when evaluating selected patients with manometry findings potentially attributable to anatomic anomalies [53,54]. Since the main target in the treatment of achalasia is the lower esophageal sphincter, high-resolution imaging of this muscular layer, as determined by mEUS, has been used to quantify esophagogastric junction muscle thickness in order to identify the phenotypes associated with outcomes after pneumatic dilation: a thickness ≥ 1.3 mm of the outer longitudinal muscle predicted mid-term recurrence with a 77.8% overall accuracy [55]. The role of mEUS has also been explored for systemic sclerosis to detect increased esophageal wall thickness involving submucosa and muscularis propria as a marker of upper gastrointestinal involvement in comparison with controls [56]. Furthermore, sub-group analysis demonstrated that dysphagia and amotility were significantly associated with the thickening of the GI walls, suggesting some insight into the pathophysiology of symptom development.

However, these observations should be interpreted cautiously, as the supporting evidence is limited by small cohort sizes and single-center designs, while direct head-to-head comparisons with standard EUS are lacking. Furthermore, the broader adoption of mEUS in benign strictures is constrained by device cost, limited probe durability, and the need for dedicated processors, which restrict availability outside high-volume centers [8].

### 3.3. Gastric Cancer

Similarly to esophageal cancer, EUS can be useful for locoregional staging and assessment of tumor invasion depth in gastric cancer, but it is not routinely recommended unless there are signs of deep submucosal invasion or the lesion is not suitable for endoscopic resection [17,57]. Currently, standard EUS shows an overall accuracy of 75% for T-staging, with high performance for distinguishing T3–T4 disease but limited reliability for superficial T categories (T1–T2) [58]. This limitation is clinically relevant because—together with lesion size, macroscopic morphology, ulceration and histologic type—the predicted tumor invasion depth helps to define the most suitable treatment for early gastric cancer (ESD vs. surgery) [17].

Some studies have highlighted that mEUS can improve the visualization of superficial wall layers and help predict whether invasion is limited to M/SM1 or deeper. In a meta-analysis of 17 studies involving 4525 early gastric cancers, Shi and Xi [59] reported that mEUS outperformed standard EUS for superficial T-staging, with 90% sensitivity and 71% specificity. Similar results emerged from the meta-analysis by Luo et al. [60], showing a negative likelihood ratio of 19% for mEUS in predicting tumor invasion depth, supporting its value as a rule-out tool for deep tumor invasion in early gastric cancer. As a consequence, pre-operative mEUS findings have been employed for treatment selection in a small cohort of early gastric cancer patients, showing appropriate selection of endoscopic treatment (for lesions invading at most the superficial submucosa), laparoscopic resection or open surgery (when deep submucosal invasion was suspected) in 100%, 91%, and 86% of cases, respectively [61]. Nevertheless, results across the literature are heterogeneous, and some authors have affirmed that the role of mEUS in early gastric cancer is debatable, as it has not been shown to increase the accuracy of predicting deep submucosal invasion when compared to conventional endoscopy or standard EUS [62,63]. Actually, a subgroup analysis of the aforementioned study by Shi and Xi had already shown relevant misclassifications, with an overstaging rate for M/SM1 disease of up to 13.3% and an understaging rate of deep SM in 29.7% of cases [59]. However, the addition of mEUS to conventional endoscopy may increase correct T-staging for high-risk phenotypes of lesions (size ≥ 2 cm, ulceration, undifferentiated histology) and reduce unnecessary surgery in 70% of early gastric cancer cases overstaged with conventional endoscopy [62].

For N-staging, mEUS diagnostic performance drops, as prospective data from a cohort of 46 patients showed high specificity (90%) but low sensitivity (17%) [64]. Even if its specificity was slightly lower (80%), standard EUS proved higher sensitivity (74%) according to the meta-analysis of Cardoso et al. [58], but the evidence was moderate as substantial heterogeneity was present across the included studies.

Overall, these results favor the use of mEUS as an adjunct to conventional endoscopy for superficial T-staging of early gastric cancer rather than as a routine exam before endoscopic/surgical therapy [59].

### 3.4. Colon and Rectal Cancer

Treatment selection for early colon and rectal cancer is primarily influenced by high-resolution endoscopy with virtual chromoendoscopy [17]. Nevertheless, standard EUS has been suggested for pre-operative staging of early colon and rectal cancer with optically suspicious features of deep submucosal invasion, which drives the choice between endoscopic resection (EMR vs. ESD) and surgery [17]. A previous meta-analysis showed comparable sensitivity of standard EUS and MRI for T1–T2 disease and higher sensitivity for T3 lesions [65], yet real-world accuracy can be substantially lower, with nearly half of cases reported as inaccurately staged [66,67,68]. Furthermore, only tumors located in the rectum and the sigmoid colon can be studied with standard echoendoscopes, whereas more proximal lesions cannot be reached [69].

In this context, mEUS can improve locoregional staging of early colon and rectal cancer, particularly when optical diagnosis is challenging for determining deep submucosal invasion or when early cancers are located in the right colon [70,71]. According to the meta-analysis of Gall et al. [72], which included 10 studies and 642 colon and rectal cancers that underwent endoscopic treatment or surgery, the overall accuracy of mEUS for T-staging was high, particularly for T1 (sensitivity 91%, specificity 98%) and T3–T4 (sensitivity 97%, specificity 90%). When only colon cancers were considered, these results were basically confirmed (Table 5), while no specific sub-analysis was made on rectal cancers only [72]. Moreover, these results must be interpreted with some limitations: there was heterogeneity in the study design and in the type of endoscopic/surgical treatment among the studies included in the analysis [72]. Further cohorts suggested that the accuracy of mEUS for detecting deep submucosal invasion of early colon and rectal cancer was particularly high for sessile and flat-type lesions [73], while it was limited when tumors were >2 cm, with villous or pedunculated-type morphology and with rectal location [73,74,75]. Similar to esophageal cancer, miniprobes can be advanced through neoplastic strictures of the colon in order to allow locoregional staging when a standard echoendoscope is unable to traverse the stricture; prospective series showed high overall accuracy for T-staging with mEUS (85%), but the assessment of deep tumor invasion remained limited (40–67%) [7,76].

Regarding N-staging, the diagnostic performance of mEUS in colon cancer showed low sensitivity (55–60%) but high specificity (>80%) [72,77]. In rectal cancer, contemporary practice indicates pelvic MRI as the preferred modality, while standard EUS is mainly recommended when MRI is contraindicated, largely because standard EUS cannot reliably assess the relationship between the tumor and the mesorectal fascia. At the same time, CT remains essential for the detection of distant metastasis [78].

In summary, mEUS can be considered a valid adjunct to standard EUS for accurate T-staging of early colon and rectal cancer when standard EUS is impractical. However, strong evidence is lacking, and most of these studies report heterogeneous results.

### 3.5. Benign Colorectal Diseases

Beyond oncology, standard EUS has been used as a transrectal assessment for benign indications, including inflammatory bowel disease, particularly for perianal Crohn’s disease complications, while its role in luminal disease activity assessment is limited by segmental feasibility restricted to the rectum and sigmoid colon [79,80]. Through-the-scope high-frequency miniprobes may partially overcome these limitations by allowing point-of-care, layer-by-layer imaging, which is complementary to the endoscopic exam. In ulcerative colitis, mEUS-based severity grading (Tsuga classification, which considers intestinal wall thickness and the interface between intestinal wall layers) showed moderate-to-good agreement with histology, particularly for mild disease (sensitivity 88%, specificity 88%) and severe disease (sensitivity 86%, specificity 96%), supporting its ability to stratify inflammatory burden beyond mucosal inspection alone [81].

In anorectal disorders, particularly in fecal incontinence, mEUS has been employed to provide detailed information on sphincter morphology alongside functional endpoints [82]. However, these applications remain exploratory and are currently supported by very limited evidence.

### 3.6. Gastrointestinal SELs

Gastrointestinal SELs are usually detected incidentally during routine endoscopy as a bulge covered with normal-appearing mucosa [83]. According to ESGE guidelines, EUS is the best tool to characterize SEL features by defining lesion size and margins, the layer of origin, internal architecture and the relationship with adjacent organs, but definitive diagnosis remains histological [16,84]. In this context, mEUS has shown high accuracy (96%) for differentiating extramural compressions from SELs [85], and because of its higher spatial resolution, it may be useful as an adjunct to standard EUS for better characterizing and defining the layer of origin of superficial SELs [16]. Although direct comparative studies between mEUS and standard EUS are lacking, a large single-center experience reported 80.1% concordance between mEUS and final histology, with particularly high agreement for lipoma (97.0%), ectopic pancreas (96.8%), and leiomyoma (91.8%), but lower accuracy for Gastrointestinal Stromal Tumors (GISTs) (63.0%), especially for lesions < 2 cm [86]. Similar results emerged in the colorectal setting, where mEUS accurately characterized the type of SEL and its layer of origin in almost 80% of patients [87]. By contrast, in a multicenter retrospective study involving 869 upper-GI SELs, mEUS achieved an overall accuracy of 70.3%, with excellent diagnostic performance for GISTs (94.8%) and poor performance for leiomyoma (9.9%) which was misclassified as GISTs in the majority of cases [82]. The apparent heterogeneity between these two large mEUS series is more likely secondary to the different designs of the studies and operator dependence rather than conflicting performances of mEUS. Khan et al. [86] reported high overall concordance in a single-center upper-GI cohort examined by two expert endosonographers using a standardized 20-MHz miniprobe protocol, a context that may favor higher agreement for ‘typical’ phenotypes and more uniform image acquisition. Conversely, Li et al. [82] evaluated a multicenter, real-world population involving non-upper-GI SELs and variable operators and equipment (12/20-MHz probes), which may amplify the misclassification rate. Importantly, both studies highlight that the main diagnostic vulnerability remains the morphological overlap of hypoechoic muscularis propria lesions. Quantitative approaches such as grayscale histogram analysis may improve discrimination between leiomyoma and GIST when conventional qualitative features overlap, yielding 85.9% sensitivity and 74.6% specificity for GIST prediction [86]. The grayscale histogram is a quantitative method that focuses on a “region-of-interest” and converts the subjective assessment of echogenicity into numeric texture features in order to capture intralesional heterogeneity on mEUS images. Because gastric GISTs more often exhibit heterogeneous internal echoes than leiomyomas, histogram-derived metrics can improve discrimination between these two hypoechoic muscularis propria lesions and mitigate interobserver variability, although results remain sensitive to image acquisition settings and region-of-interest selection [86].

Interestingly, mEUS has also been combined with submucosal saline injection of the target lesion in order to detect the “detachability” of the lesions from the muscularis propria, which was considered a practical predictor of safe complete endoscopic resection, with an overall diagnostic accuracy of 86.6%. Accordingly, a poor submucosal lifting of the SEL can be suggestive of deeper involvement and may warrant alternative approaches rather than endoscopic treatment [88].

A practical advantage that mEUS may also offer relies on the possibility of being performed during the index endoscopy, allowing prompt discrimination between intramural SEL and extramural compression and facilitating early management decisions [82].

Key limitations of mEUS include large-size SELs (>30 mm) and small SELs originating from the muscularis propria, due to restricted ultrasound penetration depth [85,87,89]. Moreover, standard EUS with tissue acquisition (FNB) and/or cross-sectional imaging remains necessary when malignancy is suspected [85]. In two retrospective studies, the detection rate of CT compared with standard EUS was 69% vs. 85.3%—with CT having a higher detection rate for SELs > 10 mm—but CT accuracy for specific diagnosis was lower (50.9% vs. 64.2%) [90,91].

In summary, mEUS can provide a rapid, point-of-care assessment for characterization and management of incidental SELs during routine endoscopy, frequently obviating the need for additional procedures, but current results are heterogeneous, and strong evidence on its diagnostic performance is lacking [85,87].

## 4. Clinical Applications of IDUS Among Biliary and Pancreatic Disorders

IDUS is performed trans-papillarily during endoscopic retrograde cholangiopancreatography (ERCP) by advancing a thin caliber probe into the bile ducts, often over a guidewire. High-frequency ultrasonography allows for a detailed assessment of the bile duct wall and subtle intraluminal lesions [92].

Within the landscape of biliopancreatic imaging, ESGE considers IDUS a useful adjunct in selected clinical scenarios, while acknowledging important limitations. Actually, IDUS has emerged as a promising tool for biliary strictures, choledocholithiasis and ampullary tumors as well as for malignancy risk stratification when standard sampling is inconclusive or as a complement to cross-sectional imaging. On the other hand, IDUS has performed less well than standard EUS for staging pancreatic malignancy and may increase the risk of pancreatitis, underscoring the need for careful patient selection [13].

### 4.1. Indeterminate Biliary Strictures (IDBSs), Cholangiocarcinoma and Benign Biliary Strictures

Over the last decade, diagnostic pathways for suspected malignant biliary strictures have progressively shifted toward EUS-guided tissue acquisition, as it provides histologic confirmation and molecular profiling, particularly when a periductal mass or suspicious lymph nodes are present and accessible [93]. However, in cases of IDBSs without an evident mass lesion, IDUSs can be considered an adjunctive imaging tool, but only when first-line exams such as brush cytology or digital single-operator cholangioscopy (DSOC) have not been diagnostic [15]. Its primary role is to identify imaging features suspicious for malignancy, thereby helping to refine risk stratification and guide therapeutic decision-making [15]. Several ultrasonographic features have been correlated with malignancy. In the earliest series, the disruption of the regular trilaminar structure of the bile duct wall emerged as the dominant feature of malignancy, whereas benign lesions were more likely to have concentric wall thickening with preserved stratification [94,95]. Asymmetric and infiltrative wall thickening or a hypoechoic mass invading the periductal tissue were also associated with malignancy [96,97,98]. Nevertheless, when the wall thickness was ≤7 mm, with a smooth and regular stratification and without external compression, malignancy was excluded with a negative predictive value of 100% [99]. Likewise, wall thickness > 7 mm and stricture length ≥ 20 mm were strongly associated with malignant obstruction [100]. Studies focused specifically on cholangiocarcinoma have also described polypoid intraductal masses at the hilum [101], and irregularity of the mucosal surface and focal thickening of the inner hypoechoic layer adjacent to the tumor have been interpreted as markers of mucosal or intramural spread for extrahepatic bile duct cancer [102].

**Figure 3:**
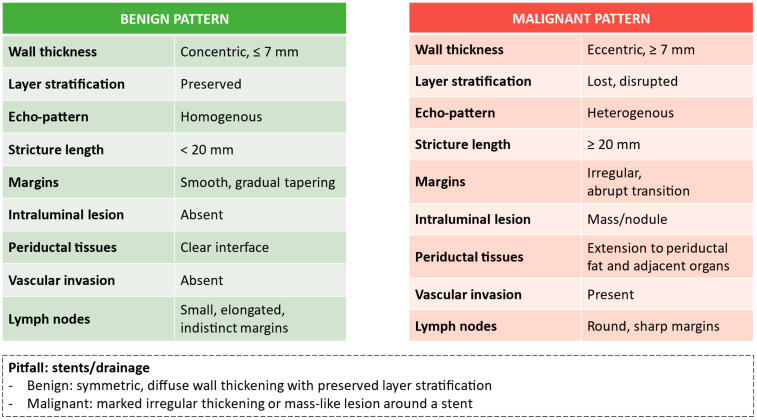
IDUS features suggestive of benign vs. malignant biliary strictures.

A summary of IDUS features suggestive of benign or malignant biliary strictures is presented in Figure 3.

Beyond descriptive morphology, several observational studies have quantified the diagnostic performance of IDUS in IDBSs. In 2015, Chen et al. [100] retrospectively analyzed 193 patients who underwent IDUS for bile duct obstruction, showing 96.9% sensitivity and 79.2% specificity for malignancy. In a larger cohort of patients with IDBSs, Meister et al. [103] showed higher specificity (near 90%) for malignancy, outperforming endoscopic trans-papillary biopsies (ETB) under fluoroscopic guidance [103]. Further prospective studies confirmed that ERCP alone or combined with ETB had lower diagnostic performance, with an overall accuracy of 67.6% at most [97,104]. However, when IDUS was added to the analysis, and both techniques characterized the stricture as malignant, the accuracy increased to 98% [98,104].

Another sampling approach with promising results consists in performing trans-papillary biopsies under IDUS guidance, whereby biopsy forceps are inserted into the bile duct in parallel to the miniprobe in order to target the portion of the stricture with a high suspicion for malignancy. This technique was evaluated by Kim et al. [105] in a prospective comparison of 67 patients, showing 90.8% accuracy. Notably, this advantage was higher for intraductal strictures, a subgroup in which fluoroscopic detection is subtle and sampling error is common; by contrast, when the target lesion is more visible (e.g., nodular morphology), both techniques performed well (95.7% vs. 91.3%), while the gain was more modest for ab-extrinsic compression (77.8% vs. 66.7%) [105].

When compared, DSOC and IDUS showed similar diagnostic performances (82.7% vs. 89.5% accuracy), while brush cytology alone was weaker (61.5% accuracy) [106]. These results support IDUS’s incremental value when DSOC is unavailable or as a complementary approach within a multimodal strategy.

Whether the diagnostic performance of IDUS varies according to stricture location remains debated. According to Chen et al. [100], IDUS achieved significantly higher specificity for proximal strictures than for distal ones (94.4% vs. 72.4%). However, this site-dependent effect was not consistently reproduced across larger series, where IDUS accuracy did not significantly differ between proximal and distal strictures [103].

For early diagnosis of cholangiocarcinoma, intraductal strategies (ERCP + IDUS ± ETB) were more accurate than cross-sectional imaging, while standard EUS showed less accuracy when compared with CT (70% vs. 79% accuracy) and performed significantly better in distal than in proximal strictures (79% vs. 57% accuracy) [107].

The available evidence on IDUS for the assessment of IDBSs is synthesized in Table 6.

Several studies have also examined the role of IDUS in the locoregional staging of cholangiocarcinoma. According to Meister et al. [103], IDUS provided reasonably accurate local T-staging (84%, 73% and 71% accuracy for T1, T2 and T3 lesions, respectively), while nodal assessment was less robust (69% accuracy for N0/N1). This result is consistent with the limited penetration depth of high-frequency IDUS probes and the inherent difficulty in reliably characterizing periductal lymph nodes [103].

IDUS has also been shown to be a valid tool for establishing the longitudinal extension of the tumor, which sometimes lacks with other imaging techniques. In the prospective study by Choi et al. [101], 30 patients with borderline resectable hilar cholangiocarcinoma underwent pre-operative CT, ERCP and IDUS before surgery: IDUS achieved 90% overall accuracy for staging the extension of the cholangiocarcinoma along the bile tree—according to the Bismuth classification—with significantly higher accuracy than CT (66.6%) and ERCP alone (60%); understaging occurred in 6.6% of cases with IDUS, compared with 26.6% for CT and 30% for ERCP [101]. More specifically, IDUS performed particularly well in detecting two of the three subtypes of intrahepatic cholangiocarcinoma (intraductal growth type and periductal infiltrating type), which were more likely to remain occult on CT or only vaguely suggested by cholangiography [101].

Within the spectrum of benign diseases, IDUS has also been applied to support the diagnosis and morphological characterization of biliary strictures related to primary sclerosing cholangitis (PSC) and IgG4-related sclerosing cholangitis (IgG4-SC). In IgG4-SC, IDUS typically shows concentric and symmetric wall thickening with smooth inner and outer margins and a relatively homogeneous internal echo-pattern; moreover, lateral mucosal lesions continuous with the hilum have been more associated with IgG4-SC [110]. In contrast, PSC strictures are more often characterized by markedly irregular luminal margins, loss of the normal four-layer wall structure and a “diverticulum-like outpouching”, which may be detected by IDUS more frequently than by ERCP with cholangiography [111]. From a diagnostic standpoint, in a prospective study of 40 patients with PSC dominant bile duct stenoses, IDUS provided high accuracy for malignancy assessment (90%), outperforming ERCP with cholangiography alone (55%) [112]. For IgG4-SC, Naitoh et al. [113] found that the wall thickness in non-stenotic bile duct segments was significantly greater than that in cholangiocarcinoma, so they proposed using a cut-off of 0.8 mm for bile duct wall thickness in non-stenotic segments, which achieved 95% sensitivity and 91% specificity for distinguishing IgG4-SC from cholangiocarcinoma.

Overall, current evidence supports the use of IDUS as a complementary tool to ERCP, as it increases the discriminative value between diagnostically challenging PSC phenotypes and for malignancy detection. On the other hand, IDUS is constrained by limited access to intrahepatic bile ducts due to probe caliber and by the need to avoid drainage-related wall thickening artifacts when possible. A source of potential pitfalls is indeed represented by catheter-induced edema/inflammation when long-term plastic and metal stents are employed, which can appear as diffuse, symmetric wall layer thickening with preserved stratification. However, a malignant pattern must be suspected in the case of marked irregular thickening or mass-like lesions around the stent [114].

A summary of IDUS differences between IgG4-SC, PSC and cholangiocarcinoma-related biliary strictures is presented in Table 7.

### 4.2. Choledocholithiasis

Another clinical application of IDUS is the evaluation of bile duct stone disease during ERCP, particularly when cholangiography is negative or equivocal and when small stones or sludge are suspected. According to Das et al. [115], adding IDUS to ERCP improved diagnostic performance primarily by increasing specificity for bile duct stone/sludge detection (96%) compared with ERCP alone (85%). More specifically, Endo et al. [116] suggested that this advantage was particularly evident for small biliary stones, which were the most likely to be missed with ERCP but detected with IDUS. As a consequence, IDUS can help avoid papillosphincterotomy when it is not required [116,117].

Some authors have also proposed using IDUS to detect residual stones/sludge that may be missed on cholangiography after bile duct toileting. In a prospective study of 70 patients, Ang et al. [118] found that, despite apparent stone extraction, IDUS still demonstrated residual small stones with a mean size of 2.2 mm in 40% of patients. Likewise, Tsuchiya et al. [119] identified residual stones/sludge in 23.7% of patients and showed that additional IDUS-guided clearance was associated with a significant reduction in biliary stone recurrence in patients who underwent ERCP + IDUS compared with ERCP alone (3.4% vs. 13.2%).

Compared with cross-sectional imaging, IDUS provided a higher diagnostic performance for detecting small biliary stones (<5 mm), with 95% sensitivity, outperforming magnetic resonance cholangiopancreatography (MRCP) (80%), CT (40%) and transabdominal ultrasound (20%) [120,121]. In this way, despite diagnostic ERCP being progressively abandoned for biliary stones, IDUS may find a role for patients with a high suspicion of choledocholithiasis.

IDUS has also been explored as a “contrast-sparing” or “lower-radiation” ERCP strategy, which can be particularly useful for infants and pregnant women. Some studies have indeed suggested that performing IDUS after bile duct toileting significantly reduced fluoroscopy time during ERCP [122,123,124]. On the other hand, when IDUS is used for stone detection, careful patient selection and pre-operative prophylaxis with rectal NSAIDs in high-risk patients should always be considered, as ERCP carries an overall adverse event rate of approximately 6–15%, with post-ERCP pancreatitis being one of the most frequent complications, and IDUS has been reported as an independent risk factor for post-ERCP pancreatitis [125].

Taken together, these findings suggest that IDUS can improve ERCP efficiency by reducing unnecessary sphincterotomy, enhancing the detection of microlithiasis, and providing confirmation of duct clearance, with potential downstream benefits on recurrence and resource utilization. However, the incremental value of IDUS is likely to be context-dependent, given the additional device costs, probe durability constraints, and variable local availability. Therefore, prospective studies incorporating cost-effectiveness analyses are warranted to define the most efficient clinical scenarios for routine IDUS adoption, particularly compared with MRCP- and standard EUS-based diagnostic pathways.

### 4.3. Ampullary Tumors

IDUS and standard EUS are used for pre-operative staging of ampullary adenomas and carcinomas. For ampullary adenomas, the presence and extent of intraductal growth are key determinants for the endoscopic strategy and may shift the indication from standard papillectomy towards radiofrequency ablation or a surgical approach [14]. Although the definitive diagnosis remains histological, sonographic features suggestive of malignant transformation have been described: a hypoechoic, inhomogeneous mass with irregular margins and signs of invasive growth [126].

In the retrospective cohort of Meister et al. [103], IDUS achieved high diagnostic performance for ampullary carcinoma detection (81% sensitivity, 90% specificity). For locoregional staging, IDUS showed good sensitivity for T1 (86%), while poorer sensitivity was observed for T3–T4 disease (60%) and for N-staging (61–66%). These findings are consistent with the meta-analysis of Ye et al. [127], which compared IDUS and standard EUS, showing similar diagnostic performance for T1 (sensitivity/specificity IDUS 89%/87% vs. standard EUS 90%/88%) and T2 (sensitivity/specificity IDUS 73%/91% vs. standard EUS 76%/91%). For N-staging, sensitivity was 61% for both techniques, but IDUS showed higher specificity than standard EUS (92% vs. 77%). This meta-analysis also assessed diagnostic performance for the detection of intraductal extension of ampullary tumors, showing that standard EUS yielded high sensitivity (79%) and specificity (88%). Only few studies included in the analysis examined IDUS performance in this field, showing higher specificity (95–99%) but variable sensitivity (respectively 14% for pancreatic duct extension and 56% for biliary duct extension) [127].

When histopathology of the endoscopic biopsies is negative, but IDUS features raise suspicion of malignancy, definitive confirmation should rely on EUS-guided tissue acquisition of the ampullary tumor and/or suspicious regional lymph nodes when accessible. End-cutting FNB needles are preferred for solid lesions [14,128].

Overall, IDUS is a valuable adjunct for the pre-operative assessment of ampullary tumors; however, the limited penetration depth of high-frequency probes constrains sensitivity for locoregional staging, particularly for nodal involvement, and histopathology remains mandatory for definitive risk stratification.

### 4.4. Other Biliary and Pancreatic Disorders

Beyond biliary strictures and choledocholithiasis, IDUS has also been explored across a wide range of biliopancreatic disorders involving periductal structures, where the high spatial resolution of catheter probes limits ultrasound penetration depth.

Portal hypertensive biliopathy represents a benign cause of biliary obstruction, where ductal narrowing arises from ectatic peri-biliary collateral veins and intraductal varices, frequently accompanied by recurrent cholangitis and bile duct stones. IDUS is particularly valuable as it can directly depict the venous structures responsible for obstruction, typically visualized as multiple hypoechoic tubular channels within the bile duct wall and/or surrounding the duct [129]. In a cohort of 377 patients who underwent ERCP for biliary abnormalities, portal hypertensive biliopathy occurred in 2.7% and was mostly secondary to extrahepatic portal vein obstruction; in this group, IDUS was able to detect biliary varices in all patients, outperforming transabdominal ultrasound, CT and MRI. Moreover, IDUS was also able to distinguish intramural varices (involving the duct’s wall veins) from periductal varices (involving collateral veins), as the former are most likely related to bile duct narrowing [129]. Clinically, this information is relevant because high-risk endoscopic interventions performed “blindly” across a stricture (e.g., balloon trawling, dilation) may precipitate severe bleeding if intramural varices are present. In these cases, IDUS can help to avoid high-risk maneuvers or assist in guiding safe biliary drainage with stenting [130,131].

Evidence on the applications of IDUS for diseases of the gallbladder is scarce, likely because meaningful assessment generally requires advancing the miniprobe through the cystic duct, which is technically demanding [132]. Even when the miniprobe is positioned in the common bile duct, the assessment of the gallbladder is constrained by the limited penetration depth of high-frequency ultrasounds. However, IDUS has recently emerged as a valuable tool for guiding endoscopic trans-papillary gallbladder drainage for acute cholecystitis, as it helped to identify the cystic duct orifice—using the portal vein as a landmark and recognizing the loss of the “partition wall” between the choledochus and the cystic duct—thereby facilitating wire access when cholangiographic guidance was difficult [10].

In early intrapancreatic applications, Furukawa et al. [133] introduced a 30-MHz 4.3 F probe into the main pancreatic duct after pancreatography, showing a very high tumor detection rate in pancreatic cancer, outperforming transabdominal ultrasound, standard EUS and CT. The typical sonographic pattern associated with pancreatic cancer was an intraductal echogenic focus with adjacent hypoechoic periductal change [134]. In a different cohort, IDUS also provided incremental diagnostic information for differentiating chronic pancreatitis, pancreatic cancer, and cystic neoplasms when abnormalities were close to the duct lumen [133]. However, no further studies have been reported recently on IDUS applications for pancreatic cancer.

IDUS has also been used to increase diagnostic yield in idiopathic recurrent pancreatitis, as it proved helpful in finding etiology in patients with negative findings on ERCP: the most common causes were microlithiasis/sludge, small polypoid intraductal lesions and ultrasonography findings associated with chronic pancreatitis [135].

A further area of interest is intraductal papillary mucinous neoplasm (IPMN), where defining ductal extent can directly influence the type of surgery and the extent of resection for high-risk lesions. In a randomized trial, Cheon et al. [136] reported that IDUS improved the accuracy of defining the lateral spread of IPMN along the main pancreatic duct compared to conventional assessment alone (standard EUS, CT, MRCP, ERCP). Accordingly, observational data suggest that IDUS may help to stratify the malignant risk of IPMN by detecting protruding intraductal lesions [137,138]. Nevertheless, the international 2024 Kyoto guidelines for IPMN surveillance do not mention IDUS and recommend standard EUS-based surveillance for branch-duct IPMN with worrisome features. Accordingly, IDUS should be considered only in selected research settings, and its routine use for IPMN management is not supported by current evidence [139].

## 5. Summary of Evidence: Comparison of mEUS and IDUS with Standard EUS and Cross-Sectional Imaging

### 5.1. mEUS and IDUS vs. Standard EUS

Across the staging of early gastrointestinal cancers (esophagus, stomach and colon-rectum), comparative evidence consistently indicates a trade-off: mEUS performs better for superficial T-staging (T1–T2), particularly when the endpoint is mucosal versus submucosal invasion, whereas standard EUS is more informative for deeper mural assessment (T3–T4) and N-staging, and it allows EUS-guided tissue acquisition of suspicious lymph nodes [12,32,33]. Despite favorable performance for T-staging in early esophageal cancer, mEUS mis-staging remains clinically relevant, particularly for lesions located near the esophagogastric junction [28,30,31]. For Barrett-associated early esophageal adenocarcinoma, both mEUS and standard EUS show only moderate discrimination between mucosal and submucosal invasion, highlighting that endoscopic resection is the preferred technique for definitive T-staging and for evaluating risk factors not evidenced by EUS [35,36,37]. In gastric cancer, standard EUS provides moderate overall T-staging accuracy, particularly less reliably for T1–T2 diseases [58], where mEUS can improve diagnostic performance for superficial T-staging assessment and may be employed as a rule-out tool for deep invasion in selected lesions [59,60]. However, mEUS misclassification rates remain clinically meaningful, and several studies reported no incremental benefit over conventional endoscopic assessment or standard EUS in routine practice, supporting its selective “adjunct” role rather than systematic use [59,62,63]. In colorectal cancer, standard EUS is mostly employed in the rectal domain and has known limitations for T1–T2 tumors with a risk of overstaging [66,67], whereas mEUS can reach more proximal locations and shows higher diagnostic performance for T-staging but weaker N-staging [72].

For SELs, mEUS and standard EUS both showed high accuracy in defining lesion size and margins, the layer of origin and internal architecture, supporting a clinically oriented risk stratification [86,87]. A key practical advantage of mEUS is that miniprobes can be deployed on demand through the working channel during the index diagnostic endoscopy, allowing single-session assessment and early decisional work-up toward surveillance, tissue diagnosis, or resection [85]. However, mEUS remains a purely imaging-based technique, and unlike standard EUS, it does not allow EUS-guided FNB; therefore, histologic confirmation remains necessary for indeterminate SELs or when subsequent management depends on tumor type [16].

For pancreatobiliary diseases, IDUS and standard EUS are more complementary than interchangeable. For IDBSs, additional IDUS can increase the diagnostic yield of ERCP for detecting malignancy [98,100,103] and guide trans-papillary biopsy sampling [104,105]. Intraductal strategies (ERCP/IDUS ± ETB) appear more accurate for early cholangiocarcinoma detection, whereas standard EUS remains superior for pancreatic cancer evaluation [13,107]. IDUS can effectively support T-staging and help define the longitudinal extension of cholangiocarinoma, while N-staging is less robust because of the limited penetration depth of high-frequency ultrasounds, favoring standard EUS when periductal nodal assessment is clinically relevant [101,102,103]. For ampullary tumors, IDUS and standard EUS show similar T-staging accuracy (particularly for T1–T2) and comparable N-staging sensitivity but higher specificity for IDUS. However, when the question is about intraductal extension, standard EUS provided higher sensitivity, whereas IDUS showed high specificity but variable sensitivity [103,127].

### 5.2. mEUS and IDUS vs. Cross-Sectional Imaging (CT/MRI)

Either mEUS or standard EUS shows higher accuracy for locoregional staging of GI cancers or SELs when compared with cross-sectional imaging, because of the lower spatial resolution of CT and MRI [72,78]. However, in selected scenarios such as rectal cancer, MRI shows high-resolution imaging and provides accurate information on extramural invasion of the tumor [67]. Moreover, CT remains mandatory for distant metastasis assessment and vascular involvement, as well as being a widely available exam. In fact, before miniprobes, CT was the only widely applicable option for preoperative locoregional imaging of lesions located in the right colon because standard echoendoscopes could not routinely reach proximal locations [69].

In the pancreatobiliary setting, the relative strengths of IDUS and cross-sectional imaging are more indication-dependent. For IDBSs, intraductal strategies (ERCP/IDUS ± ETB) can outperform cross-sectional imaging for early detection of cholangiocarcinoma, whereas CT may perform better for pancreatic lesions [13,107]. For hilar cholangiocarcinoma, IDUS can help reduce understaging, particularly in infiltrative patterns that may be equivocal on CT [101]. On the other hand, CT remains essential to assess extrabiliary invasion, vascular involvement and distant metastasis, which cannot be evaluated with mEUS [13]. Finally, IDUS can outperform the sensitivity of cross-sectional imaging in selected “small-target” scenarios such as millimetric bile duct stones and portal hypertensive biliopathy, where IDUS can delineate intramural and periductal varices more effectively [121,130].

Table 8 summarizes the suggested diagnostic techniques (mEUS/IDUS, standard EUS and CT/MRI) for each disease across the GI tract and pancreatobiliary domain.

## 6. Future Perspectives

High-frequency catheter miniprobes primarily enhance near-field imaging, but most currently available systems are B-mode-based and do not provide microvascular characterization or tissue acquisition [93]. In parallel, innovations implemented on conventional EUS platforms, particularly contrast-enhanced EUS and detective flow imaging, can add functional information by depicting perfusion and fine/slow-flow vessels, which can refine the differential diagnosis and local invasion assessment in selected biliary lesions [140]. These techniques can therefore be viewed as complementary to miniprobes, allowing both near-field high-resolution imaging and microvascular enhancement of lesions across the gastrointestinal tract and pancreatobiliary diseases.

Finally, AI-assisted interpretation and quantitative texture approaches may help standardize mEUS image reading and reduce operator dependence, particularly in challenging diagnoses [141].

## 7. Conclusions

mEUS represents high-resolution ultrasound imaging that can be employed on demand during routine endoscopy when near-field resolution is decisive. The fields of application of mEUS are not limited to settings where standard EUS is impractical (e.g., staging of GI strictures or tumors located in the right colon). In early esophageal, gastric and colorectal tumors, mEUS can adequately assess early T-staging, although optical diagnosis still represents the most accurate exam that predicts deep submucosal invasion, which is the decisive point between endoscopic resection and surgical treatment [28,60,72]. However, mEUS may be a valid adjunct when optical diagnosis is challenging [70,71] or as a rule-out tool for deep invasion in selected lesions [59,60]. For small SELs, mEUS can efficiently define the layer of origin and characterize the lesion type based on internal echo-patterns, while its combined evaluation with submucosal lifting serves as a predictor for safe and complete endoscopic resection [88]. The key advantage of mEUS lies in its single-session assessment of incidental SELs without suspicion of malignancy, but it is limited when tissue sampling with FNB is required [82,87]. In pancreatobiliary diseases, IDUS has its most important application for the assessment of IDBSs [100,102] and accurately identifies subtle intraductal abnormalities such as small stones that may be missed by cholangiography [115,121].

However, several limitations must be considered. First, the high-frequency ultrasound limits penetration depth, constraining evaluation of extramural extension and regional lymph nodes of early GI cancers. Second, image quality is highly sensitive to technical factors (e.g., adequate coupling with the lesion, inadvertent wall compression), which can alter apparent layer boundaries and contribute to both over- and understaging. This operator dependence amplifies interobserver variability, especially at clinically critical thresholds such as mucosal versus superficial submucosal invasion. Regarding safety, IDUS has been associated with a higher risk of pancreatitis, reinforcing the need for careful patient selection, preventive strategies, and expert execution. Furthermore, current evidence on mEUS is heterogeneous and is still dominated by observational studies, with variable endpoints and histopathological reference standards (e.g., different thresholds for deep submucosal invasion) [8,59,60]. Finally, the cost and durability of miniprobes, together with the need for dedicated processors, continue to restrict availability outside high-volume centers and likely contribute to the lack of mEUS-specific indications in major Western guidance documents [8].

Further high-quality studies are needed to validate these encouraging findings and define appropriate indications for mEUS/IDUS in routine practice.

## Figures and Tables

**Table 1:** Miniprobe models currently available in the market for digestive endoscopy.

Model n°	Manufacturer	Scanning Method	Frequency	Working Length	Outer Diameter (Max)	Key Specifications
UM-DP12-25R UM-DP20-25R	Olympus (Japan, Tokyo)	360° radial mechanical and helical mechanical	12 MHz 20 MHz	2050 mm	2.5 mm	• 3D imaging
UM-DG20-31R		360° radial mechanical and helical mechanical	20 MHz		3.1 mm	• 0.035 inch guidewire • 3D imaging
UM-2R UM-3R		360° radial mechanical	12 MHz 20 MHz		2.4 mm	-
UM-G20-29R		360° radial mechanical	20 MHz		2.2 mm	• 0.035 inch guidewire
UM-S20-20R UM-S20-17S		360° radial mechanical		2050 mm 2150 mm	2.0 mm 1.7 mm	• Suitable for bile ducts • Compatible with balloon-sheath
UM-S30-20R UM-S30-25R			30 MHz	2050 mm	2.0 mm 2.4 mm	-
P2020 P2015 P2012	Fujifilm (Japan, Tokyo)		20 MHz 15 MHz 12 MHz	M-type: 2120 mm	2.0 mm	-
P2625 P2620 P2615 P2612			25 MHz 20 MHz 15 MHz 12 MHz	L-type: 2620 mm	2.6 mm	-
IM-02M-01 iMP-8903	InnerMed (Bühl, Germany)	360° radial mechanical	12/20 MHz 12/20 MHz	2100 mm NA	2.5 mm	-

NA—not available.

**Table 2:** Technical comparison of mEUS versus standard EUS and cross-sectional imaging.

Parameter	mEUS/IDUS	Standard EUS	CT/MRI
Frequency	High (12–30 MHz)	Low (5–10 MHz)	-
Penetration depth	Shallow (<3 cm)	Deep (>3 cm)	Whole-body
Doppler	Only B-mode	Available	-
Tissue sampling and operative procedures	Not available	Available with linear EUS	Not available
Strengths	• Early T-staging (T1–T2) • Staging for proximal colon cancer • Staging across tight strictures • Pediatric feasibility	• Advanced T-staging (T3–T4); • N-staging and vascular assessment • Tissue sampling and operative EUS-guided procedures	• Systemic staging (M) • Mesorectal invasion assessment (MRI) • Pre-operative planning
Limitations	• Limited penetration depth • Coupling artifacts	• Over-/understaging of early cancer (T1–T2)	• Inadequate assessment of GI wall layers (except MRI in the rectum)

**Table 3:** Search strategy summary.

Items	Specification
Review design	Narrative review based on a structured literature search
Date of search	Search last updated in February 2026
Databases and other sources searched	MEDLINE/PubMed, Embase, and Scopus; manual screening of the reference lists of eligible articles and relevant reviews
Search terms used (including MeSH and free-text search terms and filters)	Combinations of controlled vocabulary and free-text terms related to “miniprobe”, “high-frequency ultrasound catheter,” “catheter probe,” “high-frequency EUS,” “through-the-scope ultrasound,” “intraductal ultrasound,” and “IDUS,” together with anatomy- and disease-specific terms including “esophagus,” “Barrett,” “esophageal cancer,” “esophageal stricture,” “early gastric cancer,” “gastric cancer staging,” “colorectal cancer,” “early colorectal cancer,” “subepithelial lesion,” “SEL,” “submucosal tumor,” “inflammatory bowel disease,” “fecal incontinence,” “bile duct,” “biliary tract,” and “pancreatic duct”
Timeframe	January 1990–December 2025
Inclusion and exclusion criteria (study type, language restrictions etc.)	Inclusion criteria: systematic reviews and meta-analyses, practice guidelines and technology reviews, prospective and retrospective clinical studies, and large case series reporting feasibility, diagnostic performance, clinical impact, or safety outcomes of mEUS/IDUS; full-text articles in English Exclusion criteria: case reports, small case series with fewer than 5 patients, editorials, letters, non-human studies, and narrative reviews without original data, except when they provided essential technical information
Selection process	Titles and abstracts of retrieved records were screened independently by two investigators, followed by full-text assessment of potentially eligible studies. Disagreements were resolved by discussion and consensus.
Data synthesis	Qualitative synthesis organized by anatomical district and clinical scenario; no quantitative meta-analysis or formal risk-of-bias assessment was performed because of the narrative design and marked heterogeneity of included studies.

**Table 4:** Summary of studies assessing diagnostic performances of mEUS and standard EUS for esophageal cancer.

Reference	N	Histology	Frequency	mEUS (†) (%)	Standard EUS (†) (%)
Hasegawa et al., 1996 [32]	25	mostly SCC	15 MHz	T(*): 94/86/92 N: 56/25/80	T(*): 78/71/76 N: 50/80/67
Hünerbein et al., 1998 [29]	10	NR	12.5 MHz	T(^): NR/NR/90 N: 75/80/78	NA
Nesje et al., 2000 [33]	54	mostly SCC	20 MHz	T(§): NR/NR/87 N: 54/100/61	T(§): NR/NR/87 N: 78/75/78
Hünerbein et al., 2003 [12]	10	NR	12.5 MHz	T(^): NR/NR/90 N: NR	NR
Murata et al., 2003 [28]	145	NR	20–30 MHz	T(^): NR/NR/87 N: NR	NR
Chemaly et al., 2008 [30]	106	54 SCC, 52 AC	20–30 MHz	T(*): 62/77/74 N: NR	NA
Pech et al., 2010 [35]	43	all AC	20–30 MHz	T(*): 69/75/69 N: NR	T(*): 64/73/76 N: NR
Meister et al., 2013 [31]	143	mostly AC	20 MHz	T(§): 73/81/75 N: 76/71/74	NA

T-staging is defined as cancer with submucosal invasion vs. cancer limited to the mucosa (*), or as T1 vs. T2 vs. T3/T4 cancer (§), or as overall depth accuracy (^); (†) results are presented as “sensitivity/specificity/accuracy” of the technique. SCC—squamous cell carcinoma; AC—adenocarcinoma; EUS—endoscopic ultrasonography; mEUS—miniprobe catheter EUS; NA—not applicable; NR—not reported.

**Table 5:** Diagnostic performance of mEUS for colon cancer staging.

Staging	Sensitivity	Specificity
T1	87%	98%
T2	71%	95%
T3/T4	98%	89%
N+	54%	88%

Source: Gall et al., 2013 [72].

**Table 6:** Studies on indeterminate biliary strictures evaluated with IDUSs.

Reference	Study Design	N	IDUS Alone (†) (%)	IDUS Combined (Technique) (†) (%)
Meister et al., 2013 [103]	Retrospective	397	93/89/91	NA
Heinzow et al., 2014 [107]	Retrospective	234	93/89/91	94/89/92 (ETB)
Chen et al., 2016 [100]	Retrospective	193	97/79/88	97/97/97 (ETB/brush cytology)
Tamada et al., 2002 [95]	Prospective	62	75/94/81	NA
Domagk et al., 2002 [104]	Prospective	60	NR/NR/83	NR/NR/98 (ETB)
Stavropoulos et al., 2005 [97]	Retrospective	60	83/83/83	93/83/90 (brush cytology)
Farrell et al., 2002 [98]	Prospective	60	NA	90/93/92 (ETB)
Sacco et al., 2024 [106]	Retrospective	52	84/80/83	NA
Menzel et al., 2000 [108]	Prospective	56	91/80/89	NA
Tamada et al., 1998 [94]	Prospective	42	89/50/76	NA
Domagk et al., 2004 [109]	Prospective	33	NR/NR/88	NA
Vazquez-Sequeiros et al., 2002 [96]	Retrospective	30	89/92/90	NA

IDUS—intraductal ultrasonography; ETB—endoscopic trans-papillary biopsies; NA—not applicable; NR—not reported. (†) Results are presented as “sensitivity/specificity/accuracy” of the technique.

**Table 7:** Differences in IDUS features of bile duct strictures related to IgG4-SC, PSC and cholangiocarcinoma.

	IgG4	PSC	Cholangiocarcinoma
Wall thickness	Concentric, symmetric	Concentric, asymmetric	Asymmetric
Wall regular stratification	Present	Often absent	Absent
Echo-pattern	Homogenous	Homogenous to heterogeneous	Heterogeneous
Luminal margin	Smooth	Irregular	Irregular, intraductal mass/nodule or polypoid lesion
Extraluminal margin	Smooth	Unclear	Irregular, abrupt interruption

IgG4-SC—IgG4-related sclerosing cholangitis, PSC—primary sclerosing cholangitis.

**Table 8:** Suggested diagnostic techniques by district and disease.

Disease	mEUS/IDUS	Standard EUS	CT/MRI
Early esophageal/gastric cancer (T-staging: rule-out deep SM invasion)	★	△	✕
Early esophageal/gastric cancer (complete T-staging and N-staging)	△	★	△
Early colon cancer (T-staging: rule-out deep SM invasion)	★	✕ (right colon) △ (left colon)	✕
Early colon cancer (complete T-staging and N-staging)	★ (right colon) △ (left colon)	✕ (right colon) ★ (left colon)	△
Rectal cancer (complete T-staging and N-staging)	△	★	★ (MRI)
Gastrointestinal obstructing cancer (complete T-staging and N-staging)	★ (tight stricture, right colon)	★ (if traversable)	△
Gastrointestinal SELs	△	★	△
IDBSs	★	△	△
Hilar cholangiocarcinoma	△ (longitudinal extension)	★	★
Choledocholithiasis	△	★	★ (MRCP)
Ampullary tumors	△	★	△
Portal hypertensive biliopathy	★	△	△
Pancreatic cancer	✕	★	★

★ = best/primary; △ = useful adjunct; ✕ = not suitable; SEL—subepithelial lesion; IDBS—indeterminate biliary stricture; MRCP—magnetic resonance cholangiopancreatography.

## Data Availability

No new data were created or analyzed in this study.

## Abbreviations

The following abbreviations are used in this manuscript:

AC	adenocarcinoma
CT	computed tomography
DSOC	digital single-operator cholangioscopy
EAC	esophageal adenocarcinoma
EMR	endoscopic mucosal resection
ERCP	endoscopic retrograde cholangiopancreatography
ESD	endoscopic submucosal dissection
ESGE	European Society of Gastrointestinal Endoscopy
ETB	endoscopic trans-papillary biopsy
EUS	endoscopic ultrasonography
FNA	fine-needle aspiration
FNB	fine-needle biopsy
GI	gastrointestinal
GIST	gastrointestinal stromal tumor
IDBS	indeterminate biliary stricture
IDUS	intraductal ultrasonography
IgG4-SC	IgG4-related sclerosing cholangitis
IPMN	intraductal papillary mucinous neoplasm
mEUS	miniprobe endoscopic ultrasonography
MRCP	magnetic resonance cholangiopancreatography
MRI	magnetic resonance imaging
PSC	primary sclerosing cholangitis
SCC	squamous cell carcinoma
